# Effect of a fluorinated pyrimidine on cachexia and tumour growth in murine cachexia models: relationship with a proteolysis inducing factor

**DOI:** 10.1054/bjoc.2000.1278

**Published:** 2000-06-02

**Authors:** H J Hussey, P T Todorov, W N Field, N Inagaki, Y Tanaka, H Ishitsuka, M J Tisdale

**Affiliations:** Pharmaceutical Sciences Research Institute, Aston University, Birmingham, B4 7ET, UK; Cytostatics Group, Nippon Roche Research Center, 200 Kajiwara, Kamakura-shi, Kanagawa, 247, Japan

**Keywords:** cancer cachexia, proteolysis-inducing factor, interleukin-6

## Abstract

The fluorinated pyrimidine nucleoside, 5′-deoxy-5-fluorouridine (5′-dFUrd) has been shown to effectively attenuate the progress of cachexia in the murine adenocarcinomas MAC16 and colon 26 as well as in the human uterine cervical carcinoma xenograft, Yumoto. Although concomitant inhibition of tumour growth was observed in all three models this was not sufficient to account for the preservation of body weight. An attempt has been made to correlate the anti-cachectic activity of 5′-dFUrd with the presence of a tumour produced proteolysis-inducing factor (PIF), thought to be responsible for the development of cachexia in the MAC16 model. Two variants of colon 26 adenocarcinoma were employed, clone 20 which produces profound cachexia, and clone 5 which produces no change in body weight in recipient animals. Mice bearing the colon 26, clone 20 variant showed evidence for the presence of PIF in tumour, serum and urine, while there was no evidence for the presence of PIF in tumour or body fluids of mice bearing the clone 5 tumours. Treatment of animals bearing the clone 20 variant with 5′-dF Urd led to the disappearance of PIF from the tumour, serum and urine concomitant with the attenuation of the development of cachexia. The human cervical carcinoma, Yumoto, which also induced cachexia in recipiant animals, showed expression of PIF in tumour, serum and urine in control and vehicle-treated mice, but was absent in mice treated with 5′-dFUrd. Thus in these experimental models cachexia appears to be correlated with the presence of PIF. © 2000 Cancer Research Campaign


British Journal of Cancer (2000) **83**(1), 56–62
© 2000 Cancer Research Campaign
doi: 10.1054/ bjoc.2000.1278, available online at http://www.idealibrary.com on IDEAL®

### Effect of a fluorinated pyrimidine on cachexia and tumour growth in murine cachexia models: relationship with a proteolysis inducing factor

**HJ Hussey[1], PT Todorov[1], WN Field[1], N Inagaki[2], Y Tanaka[2], H Ishitsuka[2] and MJ Tisdale[1]**

[1]Pharmaceutical Sciences Research Institute, Aston University, Birmingham B4 7ET, UK; Cytostatics Group, Nippon Roche Research Center, 200, Kajiwara, Kamakura-shi, Kanagawa 247, Japan

**Summary** The fluorinated pyrimidine nucleoside, 5′-deoxy-5-fluorouridine (5′-dFUrd) has been shown to effectively attenuate the progress of cachexia in the murine adenocarcinomas MAC16 and colon 26 as well as in the human uterine cervical carcinoma xenograft, Yumoto. Although concomitant inhibition of tumour growth was observed in all three models this was not sufficient to account for the preservation of body weight. An attempt has been made to correlate the anti-cachectic activity of 5′-dFUrd with the presence of a tumour produced proteolysis-inducing factor (PIF), thought to be responsible for the development of cachexia in the MAC16 model. Two variants of colon 26 adenocarcinoma were employed, clone 20 which produces profound cachexia, and clone 5 which produces no change in body weight in recipient animals. Mice bearing the colon 26, clone 20 variant showed evidence for the presence of PIF in tumour, serum and urine, while there was no evidence for the presence of PIF in tumour or body fluids of mice bearing the clone 5 tumours. Treatment of animals bearing the clone 20 variant with 5′-dF Urd led to the disappearance of PIF from the tumour, serum and urine concomitant with the attenuation of the development of cachexia. The human cervical carcinoma, Yumoto, which also induced cachexia in recipiant animals, showed expression of PIF in tumour, serum and urine in control and vehicle-treated mice, but was absent in mice treated with 5′-dFUrd. Thus in these experimental models cachexia appears to be correlated with the presence of PIF. © 2000 Cancer Research Campaign

**Keywords:** cancer cachexia; proteolysis-inducing factor; interleukin-6

The fluorinated pyrimidine 5′-deoxy-5-fluorouridine (5′-dFUrd) is the intermediate metabolite of the anticancer drug capecitabine (Xeloda ™$N^4$-pentyloxycarbonyl-5′-deoxy-5-fluorocytidine), and has been shown to reverse the wasting in the colon 26 adenocarcinoma cachexia model (Tanaka et al, 1990), independent of its antiproliferative activity (Eda et al, 1991). The mechanism for this effect is not known and the mechanism by which colon 26 adenocarcinoma induces cachexia has not been fully elucidated. Various cytokines have been implicated as mediators of the process of cachexia. Of these, interleukin-6 (IL-6) has been suggested to be most important in the colon 26 model, since serum levels of this cytokine appeared to correlate with the development of cachexia (Strassman et al, 1992), and administration of anti-IL-6 monoclonal antibody, but not anti-tumour necrosis factor-α (TNF-α) monoclonal antibody, attenuated the development of weight loss and other parameters of cachexia. However, other data (Fujimoto-Ouchi et al, 1995; Soda et al, 1995) has raised doubts as to whether IL-6 alone is responsible for cachexia in this model. Although IL-6 mRNA was selectively detected at the tumour site of a cachexia-inducing (clone 20) variant of the colon 26 adenocarcinoma, but not at a non-cachexia-inducing (clone 5) variant (Yasumoto et al, 1995), serum levels of IL-6 were found to be elevated in mice transplanted with either clone, although levels were found to be 35% lower in animals bearing clone 5 than clone 20 (Fujimoto-Ouchi et al, 1995). This suggests that IL-6 is necessary, but not sufficient, for the induction of cachexia in this model.

One candidate for an additional factor is a sulphated glycoprotein of $M_r$ 24 000 elaborated by cachexia-inducing murine (Todorov et al, 1997) and human tumours (Todorov et al, 1996*a*), which has been shown to induce a state of cachexia when administered to mice (Todorov et al, 1996*a*; Cariuk et al, 1997), associated with a depletion of skeletal muscle protein, without a loss of visceral protein (Lorite et al, 1998). This effect was caused by direct catabolism of skeletal muscle proteins (Lorite et al, 1997; Todorov et al, 1997) and for this reason the material has been named proteolysis-inducing factor (PIF).

In the present investigation the effect of 5′-dFUrd on the development of cachexia and PIF levels in tumour, serum and urine has been determined in mice bearing the colon 26 adenocarcinoma, as well as in those bearing a human uterine cervical carcinoma, Yumoto, which produces a cachexia, also thought to be mediated by IL-6 (Tamura et al, 1995). In addition the effect of this fluorinated pyrimidine has been determined on cachexia induced by the MAC16 tumour for which there is no involvement of IL-6 (Mulligan et al, 1992).

*Received 10 September 1999*
*Revised 3 January 2000*
*Accepted 28 February 2000*

*Correspondence to:* MJ Tisdale

### MATERIALS AND METHODS

### Animals

Female NMRI mice, obtained from our own colony, were transplanted into the flanks with fragments of the MAC16 tumour, as described (Beck and Tisdale, 1987), when the animals reached 6–8 weeks of age. Male BALB/c × DBA/2F$_1$ mice were purchased from SLC (Hamamatsu, Japan) and transplanted subcutaneously (s.c.) with $10^6$ colon 26 adenocarcinoma cells into the right inguinal flank when they reached 5 weeks of age (Tanaka et al, 1990). Animals were inoculated with either clone 20 (cachexia-inducing) or clone 5 (which does not induce cachexia). The human uterine cervical carcinoma, Yumoto, clone 17, was transplanted s.c. into female BALB/c-*nu/nu* mice (Charles River, Japan Inc., Atsugi, Japan) when the animals were 6–7 weeks of age (Tamura et al, 1995).

### Chemicals and treatment

5′-dFUrd was obtained from Nippon Roche KK (Tokyo, Japan). The compounds were administered orally (p.o) suspended in either 0.5% carboxymethylcellulose (colon 26 and Yumoto), or liquid paraffin (MAC16), at the concentrations and time schedules shown. Control groups received vehicle alone. The group sizes comprised either six animals (colon 26) or ten animals (MAC16 and Yumoto). The fluorinated pyrimidine was administered daily from day 13 until 20 (colon 26), days 36–40 and 43–46 (Yumoto) and days 12–17 (MAC16). For MAC16, body weight and tumour volume were determined daily during the treatment. Tumour dimensions were measured by means of calipers and the volume was calculated from the formula:

$$\frac{\text{Length} \times (\text{width})^2}{2}$$

Animals were terminated when the body weight reached 75% of their starting weight.

Urine samples were collected from mice with colon 26 tumours using metabolic cages on day 13 (before treatment) and day 20 (after treatment), and from mice with Yumoto tumours on day 26 (before treatment) and day 47 (after treatment). At the end of the experiment the animals were humanely killed and serum and organ samples were collected and weighed.

### Sample preparation

Tumours were homogenized in 10 mM Tris–HCl, pH 8.0, containing 0.5 mM phenylmethylsulphonyl fluoride, 0.5 mM EGTA and 1 mM dithiothreitol (DTT). The supernatant, obtained by low speed centrifugation (4000 rpm for 15 min on a benchtop centrifuge), was precipitated with ammonium sulphate (40% w/v) with stirring at 4°C. The precipitate was removed by centrifugation (4500 rpm for 20 min) and the supernatant was concentrated against phosphate buffered saline (PBS) using a Microcon micro-concentrator containing a membrane filter with a molecular weight cut-off of $M_r$ 10 000 (Amicon Inc., Beverly, MA, USA). The supernatant was applied overnight to an affinity column containing monoclonal antibody coupled to Affi Gel Hz (Bio-Rad, Hemel Hempstead, UK (17) and active fractions were eluted with 100 mM glycine. HCl, pH 2.5, neutralized with 1 M Tris–HCl, ultrafiltered against water using an Amicon filtration cell and used for Western blotting. Serum samples were passed through a protein A-sepharose column to remove antibodies and then subjected to affinity chromatography as above. Urine was treated with ammonium sulphate (80% w/v) and stirred overnight at 4°C. The precipitated protein was recovered by centrifugation at 3000 ***g*** for 30 min, dialysed against water using an Amicon filtration cell and used for Western blotting.

**Table 1** Effect of 5′-dFUrd on food and water intake and final tumour weights in mice bearing the MAC16 adenocarcinoma[a]

| Group | Food (g 24 h$^{-1}$) | Water (ml 24 h$^{-1}$) | Tumour wet weight (g) |
|---|---|---|---|
| Non-treated | 3.51 ± 0.02 | 3.00 ± 0.61 | 0.33 ± 0.05 |
| Vehicle | 3.45 ± 0.38 | 3.20 ± 0.58 | 0.44 ± 0.04 |
| 5′-dFUrd mmole kg$^{-1}$ day$^{-1}$ | | | |
| 0.11 | 3.60 ± 0.16 | 3.27 ± 0.21 | 0.36 ± 0.03 |
| 0.22 | 3.72 ± 1.09 | 2.89 ± 0.75 | 0.35 ± 0.04 |
| 0.44 | 3.46 ± 0.09 | 2.80 ± 0.69 | 0.25 ± 0.06 |
| 0.88 | 3.40 ± 0.20 | 3.20 ± 0.29 | 0.32 ± 0.07 |

[a]Values are expressed as mean ± s.e.m. for ten animals per group.

### Western blot analysis

Samples (5 μg protein) were loaded onto sodium dodecyl sulphate polyacrylamide gels consisting of a 5% stacking gel and a 15% resolving gel (Cariuk et al, 1997). Gels were transferred to nitrocellulose membranes (Hoefer Scientific Instruments, San Francisco, CA, USA) which had been blocked with 5% Marvel in 0.15% Tween-20 in PBS at 4°C overnight. The membranes were washed once for 15 min in 0.5% Tween-20 in PBS, followed by two more washes. The membranes were incubated for 1 h at room temperature in Tween/PBS containing 1.5% Marvel and monoclonal antibody (10 μg ml$^{-1}$) (Todorov et al, 1996*b*) which had been biotinylated using the enhanced chemiluminescence (ECL) protein biotinylation module (Amersham UK). After being washed three times as above, the filters were incubated for 1 h at room temperature with streptavidin–horseradish peroxidase conjugate (Amersham) at a 1- to 1500-fold dilution followed by three 15-min washes with 0.1% Tween in PBS. Bands were detected with an ECL system (Amersham).

### Statistical analysis

Differences between groups were analysed by the Mann–Whitney *U*-test or ANOVA followed by Tuckey's test. The level of significance was regarded as $P < 0.05$.

### RESULTS

The effect of daily p.o. administration of 5′-dFUrd, suspended in liquid paraffin, on changes in body weight and tumour growth rate in mice bearing the MAC16 tumour is shown in Figure 1. Control non-tumour-bearing animals did not significantly change in weight during the time course of Figure 1 and these data are not included. There was no difference in the rate of weight loss between vehicle-treated mice and an untreated group, but a dose-related reduction in the rate of weight loss in animals treated with 5′-dFUrd, which became significant at dose levels of 0.44 and 0.88 mmol kg$^{-1}$ day$^{-1}$ (Figure 1A). There was no difference in food or water intake between the groups (Table 1). There was a small inhibition of tumour growth rate, but this was only significant at a dose level of 0.44 mmole kg$^{-1}$ day$^{-1}$ (Figure 1B) and there was no significant difference in the tumour wet weights (Table 1). The inhibition of tumour growth rate was not sufficient to account for the preservation of body weight.

*© 2000 Cancer Research Campaign*

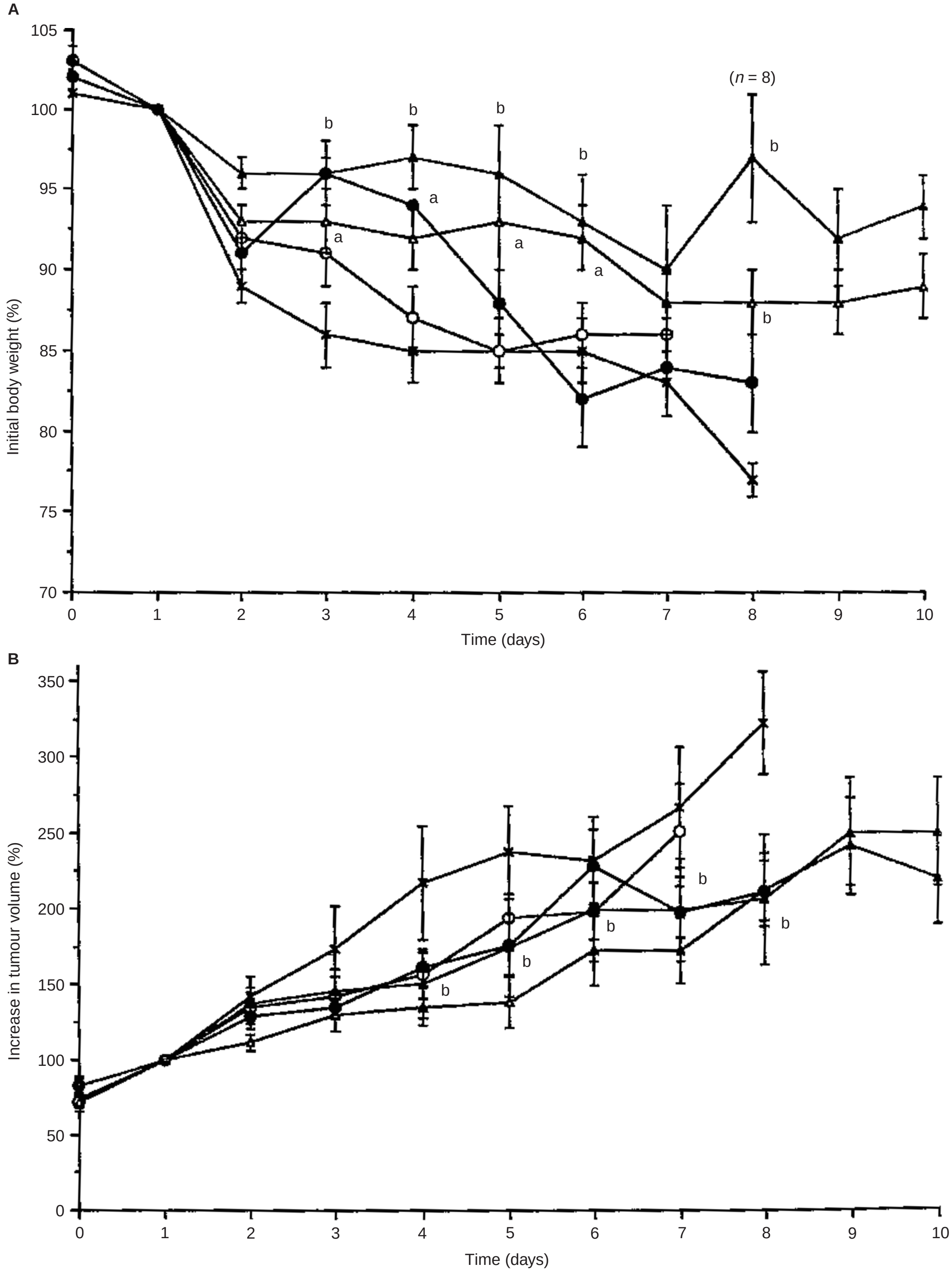

**Figure 1** The effect of daily oral administration of either vehicle (×) or 5′-dFUrd at 0.11 (○), 0.22 (●), 0.44 (△) or 0.88 (▲) mmoles kg$^{-1}$ day$^{-1}$ on body weight (**A**) and tumour volume (**B**) in mice bearing the MAC16 tumour. The results are shown as mean ± s.e.m., where $n$ = 10. Differences from vehicle-treated controls are shown as (a) $P < 0.05$; and (b) $P < 0.01$ using two-way ANOVA followed by Tuckey's test

The effect of 5′-dFUrd on tumour and body weight change in mice transplanted with murine colon 26 adenocarcinoma cells, clone 20 and clone 5 and the human cervical carcinoma, Yumoto is shown in Table 2. As previously reported (Fujimoto-Ouchi et al, 1995) colon 26 and clone 20, produced profound cachexia with a decrease in body weight arising from a loss of both adipose tissue and muscle mass, while clone 5 produced no change in body weight or muscle mass, even though the total tumour mass at day 21 was threefold greater in animals bearing the clone 5 variant than the clone 20 (Table 2). However, clone 5 did produce a significant depression of adipose tissue when compared with the non-tumour-bearing group. As in the MAC16 model 5′-dFUrd at

 © 2000 Cancer Research Campaign

**Table 2** Effect of 5′-dFUrd on tumour, body weight, adipose and gastrocnemius muscle mass between days 13 and 21 (colon 26)[a] and days 26 and 47 (Yumoto)[b]

| Tumour type | Treatment | Tumour (mg) | Body weight (g) | Adipose (mg)[c] | Gastrocnemius (mg) |
|---|---|---|---|---|---|
| Colon 26, clone 20 | Before (day 13) | 244 ± 17 | 24.9 ± 1.3 | 155 ± 30 | 136 ± 9 |
| | Non-tumour bearing (day 21) | – | 27.8 ± 1.7 | 228 ± 53 | 171 ± 17 |
| | Vehicle (day 21) | 279 ± 28[h] | 20.3 ± 3.5[j] | 24 ± 31[k] | 110 ± 11[m] |
| | 5′-dFUrd (day 21)[d] | 174 ± 26[f] | 23.5 ± 2.1[g] | 67 ± 46[f] | 125 ± 18 |
| Colon 26, clone 5 | Before (day 13) | 295 ± 85 | 26.7 ± 0.8 | 204 ± 39 | 149 ± 7 |
| | Vehicle (day 21) | 863 ± 184[i] | 27.2 ± 1.1 | 118 ± 19[k] | 157 ± 11 |
| | 5′-dFUrd (day 21)[d] | 432 ± 114[f] | 25.5 ± 0.4 | 129 ± 54[j] | 157 ± 23 |
| Yumoto, clone 17 | Before (day 26) | 470 ± 168 | 20.6 ± 1.2 | 15.4 ± 9.6 | 88.5 ± 19.8 |
| | Non-tumour bearing (day 47) | – | 21.6 ± 1.1 | 49.1 ± 20.3 | 112.8 ± 13.0 |
| | Vehicle (day 47) | 1157 ± 294 | 19.6 ± 1.9[k] | 11.1 ± 8[l] | 88.2 ± 11.5[n] |
| | 5′-dFUrd (day 47)[d] | 713 ± 264[f] | 20.8 ± 1.5 | 19.8 ± 11.5 | 97.6 ± 8.8[f] |
| | 5′-dFUrd (day 47)[e] | 655 ± 175[f] | 21.1 ± 1.4[f] | 27.3 ± 25.6[f] | 99.2 ± 12.3[f] |

[a] Results are expressed as mean ± s.d. for six mice per group; [b] Results are expressed as mean ± s.d. for ten mice per group; [c] Epididymal adipose tissue for colon 26 and periovarian for Yumoto; [d] 0.5 mmol $kg^{-1}$ $day^{-1}$; [e] 1.0 mmol $kg^{-1}$ $day^{-1}$; [f] $P < 0.05$ compared with vehicle-treated group; [g] $P = 0.05$ from vehicle-treated group; [h] $P = 0.05$ from day 13; [i] $P = 0.004$ from day 13; [j] $P = 0.01$ from non-tumour bearing group; [k] $P = 0.003$ from non-tumour bearing group; $P = 0.0002$ from non-tumour bearing group; [m] $P = 0.007$ from non-tumour bearing group; [n] $P = 0.002$ from non-tumour bearing group.

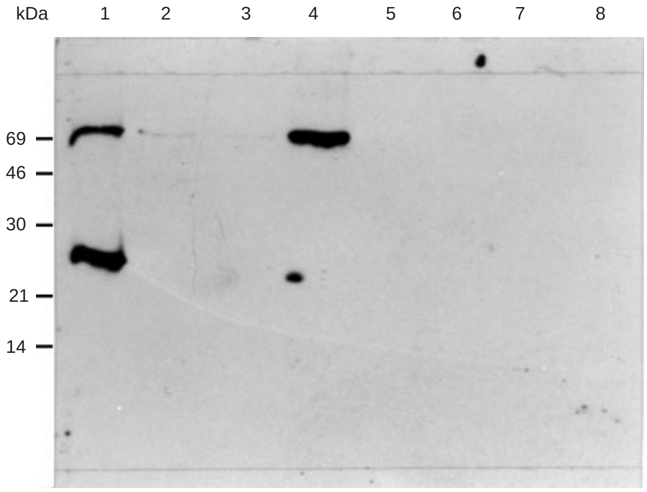

**Figure 2** Immunoblot of urine from mice bearing colon 26 adenocarcinoma. Lane 1, clone 20, day 13; lane 2, clone 5, day 13; lane 3, empty; lane 4, clone 20, day 20, vehicle; lane 5, clone 20, day 20, 0.5 mmole $kg^{-1}$ $day^{-1}$ 5′-dFUrd; lane 6, clone 5, day 20 vehicle; lane 7, clone 5, day 20, 0.5 mmole $kg^{-1}$ $day^{-1}$ 5′-dFUrd; lane 8, empty

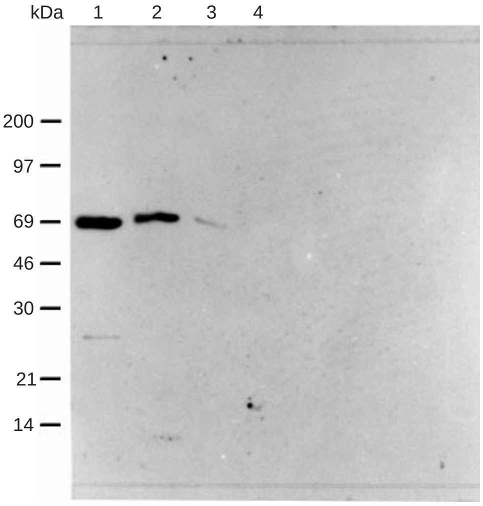

**Figure 3** Immunoblot of urine from mice bearing Yumoto tumour. Lane 1, day 26; lane 2, day 47, vehicle; lane 3, day 47, 0.5 mmole $kg^{-1}$ $day^{-1}$ 5′-dFUrd; lane 4, day 47, 1.0 mmole $kg^{-1}$ $day^{-1}$ 5′-dFUrd

0.5 mmole $kg^{-1}$ $day^{-1}$ suppressed the loss of body weight, adipose tissue and gastrocnemius muscle in animals bearing clone 20 tumours, while reducing tumour burden in mice bearing both the clone 20 and clone 5 variants. In the Yumoto model 5′-dFUrd also effectively suppressed tumour development and attenuated the development of cachexia at the higher dose. Previous studies with 5′-dFUrd have indicated that the recovery from cachexia is not the result of the reduction in tumour size (Eda et al, 1991).

To determine whether 5′-dFUrd had any effect on factors that may be responsible for the cachexia, urine, serum and tumour samples were probed by Western blotting for changes in the production of PIF. The results from urine samples of mice bearing the colon 26 tumour are shown in Figure 2. We have previously reported that PIF occurs in a free from of $M_r$ 24 000 and an albumin bound form of $M_r$ 69 000 (Todorov et al, 1996*a*). Animals bearing clone 20 tumours showed evidence for the excretion of both forms of PIF on day 13 (Figure 2, lane 1) and this was also seen in vehicle-treated control mice on day 20 (Figure 2, lane 4). Treatment with 5′-dFUrd (Figure 2, lane 5) resulted in the complete abolition of the excretion of both forms of PIF. In contrast with these results in mice bearing the clone 20 variant, mice bearing colon 26, clone 5, showed no evidence for the excretion of either form of PIF either on day 13 (Figure 2, lane 2) or day 20 (Figure 2, lanes 6 and 7). A Western blot of urine from mice bearing the Yumoto tumour is shown in Figure 3. Again control animals at day 26 (Figure 3, lane 1) and vehicle treated controls at day 47 (Figure 3, lane 2) showed evidence for the excretion of PIF, although in this case only the albumin bound form was observed. Treatment with 5′-dFUrd at the lower dose level (Figure 3, lane 3), caused a substantial reduction in the intensity of the $M_r$ 69 000 band, while at the higher dose level it was completely absent (Figure 3, lane 4).

*© 2000 Cancer Research Campaign* 

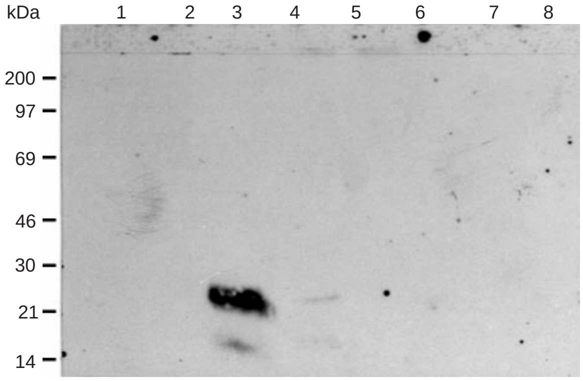

**Figure 4** Immunoblot of serum from mice bearing colon 26 adenocarcinoma. Lane 1, non-tumour-bearing, day 13; lane 2, non-tumour-bearing, day 20; lane 3, clone 20, day 13; lane 4 empty; lane 5, clone 20, day 20, 0.5 mmole $kg^{-1}$ $day^{-1}$ 5′-dFUrd; lane 6, clone 5, day 13; lane 7, clone 5, day 20 vehicle; lane 8, clone 5, day 20, 0.5 mmole $kg^{-1}$ $day^{-1}$ 5′-dFUrd;

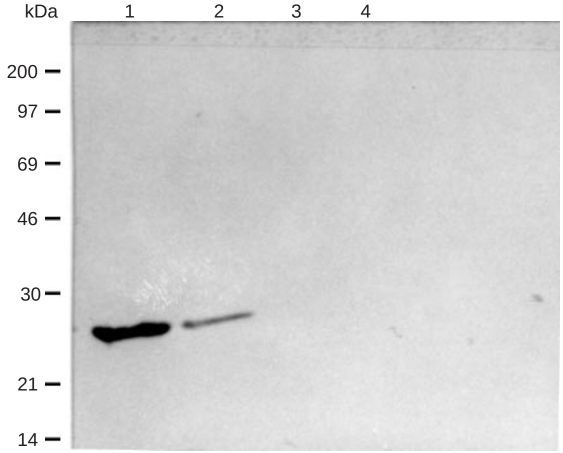

**Figure 5** Immunoblot of serum from mice bearing the Yumoto tumour. Lane 1, day 47 vehicle; lane 2, day 47, vehicle; lane 3, day 47, 5′-dFUrd 0.5 mmole $kg^{-1}$ $day^{-1}$; lane 4, day 47, 5′-dFUrd, 1.0 mmole $kg^{-1}$ $day^{-1}$

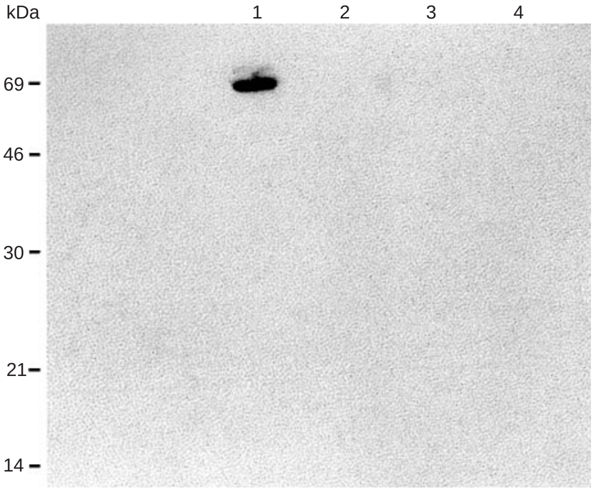

**Figure 6** Immunoblot of colon 26 adenocarcinoma. Lane 1, clone 20, day 16; lane 2, clone 5; lanes 3 and 4, clone 20, day 23 after 5′-dFUrd at 0.3 mmole $kg^{-1}$ $day^{-1}$ (lane 3) and 0.5 mmole $kg^{-1}$ $day^{-1}$ (lane 4)

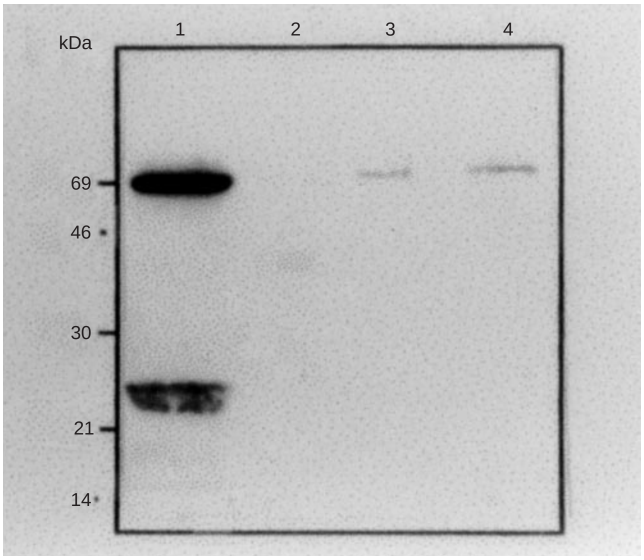

**Figure 7** Immunoblot of Yumoto tumour. Lane 1, untreated; lane 2, empty; lane 3, 5′-dFUrd 0.5 mmole $kg^{-1}$ $day^{-1}$; lane 4, 5′-dFUrd 1.0 mmole $kg^{-1}$ $day^{-1}$

Similar results were obtained using serum from tumour-bearing mice (Figures 4 and 5). No immunoreactive bands were observed in serum from non-tumour-bearing mice (Figure 4, lanes 1 and 2), while serum from mice bearing colon 26, clone 20 showed a strong positive band at $M_r$ 24 000 (Figure 4, lane 3), which was completely absent in mice treated with 5′-dFUrd (Figure 4, lane 5). As for urine, serum from mice bearing the clone 5 variant showed no evidence for the presence of PIF in the presence or absence of 5′-dFUrd (Figure 4, lanes 6–8). Serum from mice bearing the Yumoto tumour also showed evidence for the $M_r$ 24 000 form of PIF (Figure 5, lanes 1 and 2), and this completely disappeared from the serum of mice treated with 5′-dFUrd (Figure 5, lanes 3 and 4). It is not known why the free form of PIF is present in the serum, while the albumin bound form is present in the urine, but may be related to transport through the kidneys.

When colon 26 and Yumoto tumours were probed for the presence of PIF the results paralleled that found in serum and urine (Figures 6 and 7). Thus colon 26, clone 20, showed evidence for expression of the albumin bound form of PIF (Figure 6, lane 1) and this was not evident in clone 5 tumours (Figure 6, lane 2). Both forms of PIF disappeared from clone 20 tumours treated with 5′-dFUrd (Figure 6, lanes 3 and 4). Yumoto tumours also showed evidence for the presence of PIF (Figure 7, lane 1), while this was absent from tumours treated with 5′-dFUrd (Figure 7, lanes 3 and 4).

### DISCUSSION

This study shows that 5′-dFUrd is an effective inhibitor of cachexia in three animal models tested; MAC16 and colon 26 as well as the xenotransplanted tumour, Yumoto. Capecitabine, a prodrug of 5′-dFUrd also showed anti-cachectic activity in the MAC16 model (results not shown). Anti-cachectic activity in the MAC16 model was seen at concentrations producing minimal inhibition of tumour growth. The MAC16 tumour is generally refractory to most cytostatic drugs including 5-FU (Double and

© 2000 Cancer Research Campaign

Bibby, 1989). This suggests that 5′-dFUrd may interfere with the mediator(s) of cachexia in these animal models.

Knowledge of the mechanism by which tumours induce a cachectic state is essential to the development of effective inhibitors. While cytokines such as TNF-α (Beutler and Cerami, 1986), IL-6 (Strassman et al, 1995), IL-1 (Moldawer et al, 1988), interferon-γ (IFN-γ) (Matthys et al, 1991) and leukaemia inhibitory factor (LIF) (Mori et al, 1991) have been implicated as mediators of the cachectic process in some animal models there is little data to substantiate their playing a major role in human cancer cachexia. In contrast, in a study of 47 patients with carcinoma of the lung, pancreas, breast, ovary, colon, rectum and liver (Cariuk et al, 1997), PIF was detected in the urine of all patients with a weight loss greater than 1 kg $month^{-1}$, but was not present in the urine of patients with the same type of tumour who were weight-stable. Patients with weight loss due to surgery, sepsis, multiple injuries, burns, acute pancreatitis and sleeping sickness showed no evidence for excretion of PIF in the urine. This suggests that PIF may play a more important role in human cancer cachexia than the cytokines.

Thus although there is considerable evidence to support a role for IL-6 in the development of cachexia in the colon 26 adenocarcinoma model (Strassman et al, 1995), most workers suggest that it is unlikely to act alone, but may induce or work in synergy with other cachectic factors (Fujiki et al, 1997; Fujimoto-Ouchi et al, 1995; Soda et al, 1995). This study provides support that this factor may be PIF, both in the colon 26 and Yumoto models. Thus in colon 26, clone 20, which induces cachexia, there is evidence for the presence of PIF in tumour, serum and urine, while in clone 5, which does not induce cachexia, there is no evidence for PIF in either the tumour serum or urine. Interestingly clone 5 does cause loss of adipose tissue, suggesting production of a lipid mobilizing factor (LMF) in the absence of PIF (Todorov et al, 1998). The Yumoto tumour also shows the presence of PIF, as does serum and urine from mice transplanted with this tumour. Further evidence to support a role of PIF in the development of cachexia in these models is provided by the anti-cachectic agent 5′-dFUrd. Thus in mice bearing colon 26, clone 20 tumours, treated with 5′-dFUrd there was a complete disappearance of PIF from tumour, serum and urine coincident with the attenuation of the development of cachexia. Similar data was obtained from mice bearing Yumoto tumours.

These results suggest that 5′-dFUrd may interfere with the biosynthesis of PIF and that this may be responsible for the anti-cachectic activity. Although 5′-dFUrd also causes suppression of tumour growth previous studies have shown that the anti-cachectic effect is distinct from the antiproliferative activity. Thus 5′-dFUrd immediately reversed the development of cachexia even at doses allowing tumour growth (Eda et al, 1991). In sublines of colon 26 which were resistant to the anti-cachectic effect of 5′-dFUrd, growth inhibition was still observed (Eda et al, 1991). This suggests that 5′-dFUrd may inhibit the production of substances triggering the development of cachexia.

Very few agents have been demonstrated to have true anti-cachectic activity. However, one such agent, eicosapentaenoic acid (EPA) has been shown to attenuate the development of cachexia, both in the MAC16 model (Beck et al, 1991) and in patients with unresectable pancreatic cancer (Wigmore et al, 1996). The effect of EPA in counteracting skeletal muscle protein degradation has been attributed to the ability to antagonise the action of PIF (Lorite et al, 1997). This suggests that future agents aimed at alleviating cancer cachexia should be directed more towards the action of PIF than the cytokines.

This study has shown 5′-dFUrd to display profound anti-cachectic activity in two murine and one human model. Further studies will be directed towards the evaluation of this agent and capecitabine in human cancer cachexia.

### ACKNOWLEDGEMENT

This work has been supported by Nippon Roche.

### REFERENCES

Beck SA and Tisdale MJ (1987) Production of lipolytic and proteolytic factors by a murine tumor-producing cachexia in the host. *Cancer Res* **47**: 5919–5923.

Beck SA, Smith KL and Tisdale MJ (1991) Anticachectic and antitumor effect of eicosapentaenoic acid and its effect on protein turnover. *Cancer Res* **51**: 6089–6093.

Beulter B and Cerami A (1986) Cachectin and tumor necrosis factor as two sides of the same biological coin. *Nature* **320**: 584–588.

Cariuk P, Lorite MJ, Todorov PT, Field WN, Wigmore SJ and Tisdale MJ (1997) Induction of cachexia in mice by a product isolated from the urine of cachectic cancer patients. *Br J Cancer* **76**: 606–613.

Double JA and Bibby MC (1989) Therapeutic index: a vital component in selection of anticancer agents for clinical trial. *J Natl Cancer Inst* **81**: 988–994.

Eda H, Tanaka Y and Ishitsuka H (1991) 5′-Deoxy-5-flurouridine improves cachexia by a mechanism independent of its antiproliferative action in colon 26 adenocarcinoma-bearing mice. *Cancer Chemother Pharmacol* **29**: 1–6.

Fujiki F, Mukaida N, Hirose K, Ishida H, Harada A, Ohno S, Bluethmann H, Kawakami M, Akiyama M, Sone S and Matsushima K (1997) Prevention of adenocarcinoma colon 26-induced cachexia by interleukin 10 gene transfer. *Cancer Res* **57**: 94–99.

Fujimoto-Ouchi K, Tamura S, Mori K, Tanaka Y and Ishitsuka H (1995) Establishment and characterization of cachexia-inducing and non-inducing clones of murine colon 26 carcinoma. *Int J Cancer* **61**: 522–528.

Lorite MJ, Cariuk P and Tisdale MJ (1997) Induction of muscle protein degradation by a tumour factor. *Br J Cancer* **76**: 1035–1040.

Lorite MJ, Thompson MG, Drake JL, Carling G and Tisdale MJ (1998) Mechanism of muscle protein degradation induced by a cancer cachectic factor. *Br J Cancer* **78**: 850–856.

Matthys P, Heremans H, Opdenakker G and Billiau A (1991) Anti-interferon-γ antibody treatment, growth of Lewis lung tumours in mice and tumour-associated cachexia. *Eur J Cancer* **27**: 182–187.

Moldawer LL, Anderson C, Gelin J and Lundholm KG (1988) Regulation of food intake and hepatic protein synthesis by recombinant-derived cytokines. *Am J Physiol* **254**: G450–G456.

Mori M, Yamaguchi K, Honda S, Nagasaki K, Ueda M, Abe O and Abe K (1991) Cancer cachexia syndrome developed in nude mice bearing melanoma cells producing leukemia inhibitory factor. *Cancer Res* **51**: 6656–6659.

Mulligan HD, Mahony SM, Ross JA and Tisdale MJ (1992) Weight loss in a murine cachexia model is not associated with the cytokines tumour necrosis factor-α or interleukin-6. *Cancer Lett* **65**: 239–243.

Soda K, Kawakami M, Kashii K and Miyata M (1995) Manifestations of cancer cachexia induced by colon 26 adenocarcinoma are not fully ascribable to interleukin-6. *Int J Cancer* **62**: 332–336.

Strassman G, Fong M, Kenney JS and Jacob CO (1992) Evidence for the involvement of interleukin 6 in experimental cancer cachexia. *J Clin Invest* **89**: 1681–1684.

Tamura S, Fujimoto-Ouchi K, Mori K, Endo M, Matsumoto T, Eda H, Tanaka Y, Ishitsuka H, Tokita H and Yamaguchi K (1995) Involvement of human interleukin 6 in experimental cachexia induced by a human uterine cervical carcinoma xenograft. *Clin Cancer Res* **1**: 1353–1358.

Tanaka Y, Eda H, Fujimoto K, Tanaka T, Ishikawa T and Ishitsuka H (1990) Anticachectic activity of 5′-deoxy-5-fluorouridine in a murine tumor cachexia model, colon 26 adenocarcinoma. *Cancer Res* **50**: 4528–4532.

Todorov P, Cariuk P, McDevitt T, Coles B, Fearon K and Tisdale M (1996*a*) Characterization of a cancer cachectic factor. *Nature* **379**: 739–742.

Todorov PT, McDevitt TM, Cariuk P, Coles B, Deacon M and Tisdale MJ (1996*b*) Induction of muscle protein degradation and weight loss by a tumor product. *Cancer Res* **56**: 1256–1261.

© 2000 Cancer Research Campaign

Todorov PT, Deacon M and Tisdale MJ (1997) Structural analysis of a tumor-produced sulfated glycoprotein capable of initiating muscle protein degradation. *J Biol Chem* **272**: 12279–12288.

Todorov PT, McDevitt TM, Meyer DJ, Ueyama H, Ohkubo I and Tisdale MJ (1998) Purification and characterization of a tumor lipid-mobilizing factor. *Cancer Res* **58**: 2353–2358.

Wigmore SJ, Ross JA, Falconer JS, Plester CE, Tisdale MJ, Carter DC and Fearon KCH (1996) The effect of polyunsaturated fatty acids on the progress of cachexia in patients with pancreatic cancer. *Nutrition* **12**: S27–S30.

Yasumoto K, Nukaida N, Harada A, Kuno K, Akiyama M, Nakashima E, Fujioka N, Mai M, Kasahara T, Fujimoto-Ouchi K, Mori K, Tanaka Y and Matsushima K (1995) Molecular analysis of the cytokine network involved in cachexia in colon 26 adenocarcinoma-bearing mice. *Cancer Res* **55**: 921–927.

*© 2000 Cancer Research Campaign*
